# Parameters Associated with the Required Drug Dose of Intravenous Immunoglobulin in Stable Chronic Inflammatory Demyelinating Polyradiculoneuropathy

**DOI:** 10.3390/neurolint15010027

**Published:** 2023-03-10

**Authors:** Ludger Feyen, Christina Schaub, Julian Zimmermann, Louisa Nitsch

**Affiliations:** 1 Department of Diagnostic and Interventional Radiology, Helios Klinikum Krefeld, 27664 Krefeld, Germany; 2 Faculty of Health, School of Medicine, University Witten/Herdecke, 58455 Witten, Germany; 3 Department of Diagnostic and Interventional Radiology, Helios University Hospital Wuppertal, 60865 Wuppertal, Germany; 4 Department of Neurology, University Hospital Bonn, Venusberg-Campus 1, 53127 Bonn, Germany

**Keywords:** CIDP, IVIg, maintenance therapy, dosing

## Abstract

Background: Intravenous immunoglobulin (IVIg) is efficient and one of very few treatment options for patients with chronic inflammatory demyelinating polyradiculoneuropathy (CIDP). However, finding the optimal dose of IVIg for individual CIDP patients remains challenging. The dose of IVIg needs to be adjusted individually. Considering the high healthcare costs of IVIg therapy, the overtreatment of some patients seen in placebo studies and the shortage of IVIg we recently experienced, as well as identifying factors associated with the required dose of IVIg in maintenance treatment, is extremely important. Thus, in this retrospective study, we analyze characteristics of patients with stable CIDP, which are associated with the required drug dose. Methods: 32 patients with stable CIDP treated with IVIg between July 2021 and July 2022 were identified from our database and included in this retrospective study. Patients’ characteristics were registered, and parameters were identified that were associated with the IVIg dose. Results: Age, cerebrospinal fluid protein elevation, disease duration, delay between symptom onset/diagnosis, Inflammatory Neuropathy Cause and Treatment (INCAT) score, and Medical Research Council Sum Score (MRC SS) were significantly associated with the required drug dose. In addition, an association of age, sex, elevated CSF protein, time interval between symptom onset and diagnosis, and the MRC SS with the required IVIg dose could be demonstrated in the multivariable regression analysis. Conclusions: Our model, which is based on routine parameters that are simple to address in the clinical practice, can be useful in adjusting the IVIg dose in patients with stable CIDP.

## 1. Background

Chronic inflammatory demyelinating polyradiculoneuropathy (CIDP) is an acquired, chronic autoimmune disorder of the peripheral nervous system, which targets the myelin in peripheral nerves. The incidence ranges from 0.33–2.81 per 100,000 [1]. Therefore, CIDP is a rare disease, but it is the most common autoimmune peripheral nerve disorder. It is mediated by cellular and humoral mechanisms, although specific antigens have not been identified [2].

It is a heterogeneous disease. CIDP can affect children, as well as patients beyond the eighth decade of life [1]. Approximately 50% of patients have a typical disease course, which is defined as over two months progressive or relapsing-remitting, as well as symmetric motor weakness with absent or diminished reflexes accompanied with sensory deficits [3]. However, the clinical presentation of CIDP is variable, and beside typical CIDP, several variants exist [4]. Cranial nerve involvement can occur, but it is rather rare. Some CIDP patients present with an acute onset, which is indistinguishable from Guillain–Barre Syndrome (GBS). Diagnosis is made using a combination of clinical parameters, electrodiagnostic studies, and laboratory findings. Guidelines for diagnosis and treatment have been revised several times. The most recent update is the European Academy of Neurology/Peripheral Nerve Society guideline 2021 [5].

Intravenous immunoglobulins (IVIg) are efficient and one of very few treatment options for patients with CIDP. Although new therapies for CIDP are being investigated in clinical trials [6], up to now, IVIg induction and maintenance treatment beside subcutaneous immunoglobulin (SCIg), steroids, and plasma exchange represents the first line treatment [7]. Most patients have a favorable response to one of the first-line treatments.

**Figure 1:**
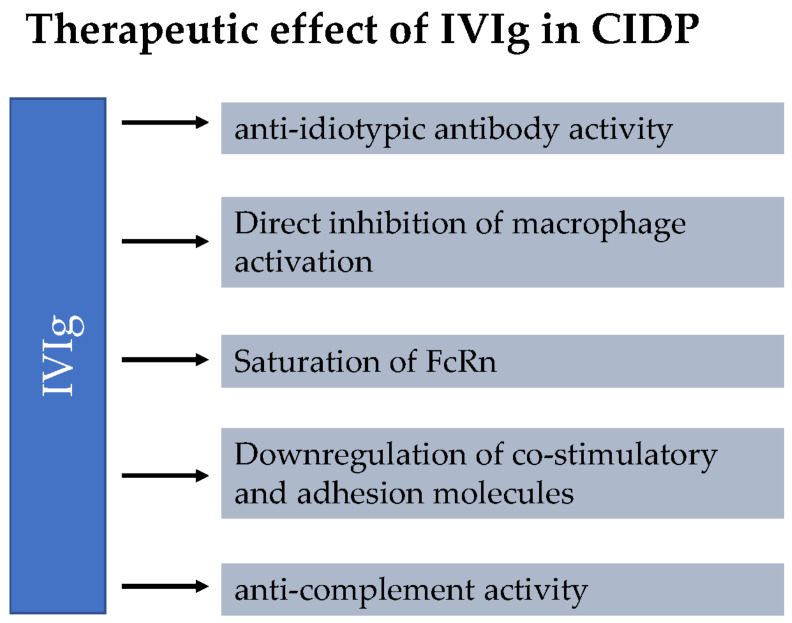
Mechanisms of action of intravenous immunoglobulins (IVIg) in chronic inflammatory demyelinating polyradiculoneuropathy (CIDP). It is hypothesized that the therapeutic effect of IVIg in CIDP is mediated by multiple mechanisms. IVIg treatment inhibits autoantibodies to bind to their antigen (anti-idiotypic effect of IVIg). The saturation of the neonatal Fc receptor (FcRn) leads to reduction of the circulating antibodies. In addition, IVIg seems to have a direct inhibitory effect on macrophage activation and interferes with activation of co-stimulatory molecules and cell-adhesion molecules. IVIg therapy leads to inhibition of complement activity, which is important for antibody-mediated cytotoxicity and macrophage activation.

IVIg is extracted from the plasma of >1000 blood donors. The exact mechanisms of action of IVIg in CIDP remain unclear and are the focus of ongoing research. It is assumed that the therapeutic effect of IVIg in CIDP is mediated by several mechanisms [2]. An overview of these mechanisms is provided in Figure 1. IVIg treatment inhibits autoantibodies to bind to their antigen. The so-called anti-idiotypic effect of IVIg was demonstrated in several studies [2]. Treatment with IVIg also reduces the level of circulating antibodies. This seems to be related to saturation of the neonatal Fc receptor (FcRn). FcRn binds IgG and reduces lysosomal degradation by transporting IgG back to the cell surface. Thus, IgG is able to re-enter circulation. Supraphysiological IgG levels after IVIg administration saturate the FcRn, leading to degradation of endogenous IgG, rather than recycling. Furthermore, IVIG therapy leads to inhibition of complement activity, which is important for antibody-mediated cytotoxicity and macrophage activation [2]. In addition, IVIg seems to have a direct inhibitory effect on macrophage activation and interferes with activation via co-stimulatory molecules and cell-adhesion molecules.

The use of IVIg is increasing worldwide for all approved indications as access to IVIg becomes available in more countries and as more patients are diagnosed with rare diseases. [8,9]. However, IVIg is a limited resource. Only a certain number of countries can provide the global demand. We recently experienced a supply shortage during the SARS-CoV-2 pandemic due to limitations in manufacturing and source plasma collection [9]. Local and global trade restrictions reduced the number of blood donations. In addition, the impact of the pandemic on pharmaceutical manufacturing and distribution affected the delivery of the final drug product to the patient [9].

Regarding side effects, IVIg infusions can cause minor side effects, such as headache, nausea, fever, rash, and transient hypertension [10]. Rarely, patients can develop signs of meningeal irritation. In contrast, serious side-effects are very rare [11].

Although there is strong evidence supporting the use of IVIg in treating CIDP, finding the optimal dose of IVIg for individual CIDP patients remains challenging [12,13,14,15]. The usual induction treatment is 2 g/kg IVIg in CIDP patients. The Progress in Chronic Inflammatory Demyelinating polyneuropathy (ProCid) study randomized patients after receiving the standard IVIg induction dose to either 0.5, 1.0 or 2.0 g/kg maintenance doses every three weeks and demonstrated that 1 g/kg IVIg every three weeks is efficacious as a maintenance treatment [16]. Guidelines recommend that maintenance dose of IVIg may need to be adjusted individually. If the patient is clinically stable, it is recommended to check periodically whether the IVIg dose can be reduced [7].

Therefore, dose reduction or treatment discontinuation is currently the only possibility to evaluate the maintenance treatment in stable CIDP patients. There is limited evidence on how long treatment with IVIg should be performed. Therefore, there is a risk of overtreatment in stable patients, resulting in high health-care costs and exposing the patients to the rare but unnecessary risk of side effects of the medication. Among patients in former CIDP trials, >50% patients randomized to placebo did not deteriorate and were not dependent on IVIg to stabilize the disease [6]. A recent study investigated discontinuation attempts of IVIg in stable CIDP patients [17]. An amount of 41% of the patients remained stable during withdrawal of IVIg for 24 weeks compared to 58% in the continuation group and 28% in the withdrawal group remained stable up to 52 weeks. In the patients with relapse after stopping the IVIg infusion, 94% of the patients restabilized within 12 weeks after re-treatment with IVIg. Taken together, the study showed that discontinuation of IVIg in stable CIDP patients is safe, and some patients may be overtreated. Hence, it is very important to elaborate parameters to identify CIDP patients whose IVIg therapy can be reduced without risk of clinical deterioration, patients who require higher doses of IVIg, and in whom IVIg reduction should be performed with greater caution. However, CIDP is a heterogenous disease, and established biomarkers for disease activity do not exist [17,18]. Thus, in the lack of biomarkers to assess activity of CIDP under IVIG treatment, the aim of the present study was to identify baseline variables and clinical parameters that are associated with the required medication dose in our cohort of stable CIDP patients. We aimed to find factors associated with a higher dose of IVIg in stable CIDP patients.

## 2. Methods

### 2.1. Study Sample and Data Collection

32 patients with the diagnosis of CIDP treated with IVIg between July 2021 and July 2022 were identified from our database and included in this retrospective study. CIDP patients are monitored every three to twelve months in our department. Reducing of the dose is performed in patients with stable clinical findings and increasing of the dose evaluated in patients with deterioration. Only patients who had been treated with IVIg for more than twelve months and who were receiving a stable dose of IVIg for more than six months were included in this study.

Patients’ characteristics, including age, sex, relevant co-morbidity (restricted to diabetes and obesity), and monoclonal gammopathy of undetermined significance (MGUS) were registered. In addition, the results of the lumbar puncture (cerebrospinal fluid (CSF) protein elevation and pleocytosis) performed at diagnosis were analyzed. Furthermore, if they received IVIg monotherapy, initial presentation of the CIDP (GBS-like manifestation, relapsing form, cranial nerve involvement, initial manifestation upper/lower extremity, or both), disease duration, delay between symptom onset/diagnosis, and last documented clinical parameters (Inflammatory Neuropathy Cause and Treatment (INCAT) Score, Medical Research Council Sum Score (MRC SS)) were registered.

The INCAT score and the MRC SS were developed to evaluate patients with inflammatory polyneuropathies. Both scores have been validated in multiple clinical trials and play a role in daily clinical practice to assess disease severity and progression and to evaluate the treatment response. The INCAT score comprises two parts, the arm and the leg score. Each part is scored from 0 to 5 points, resulting in a score between 0 and 10 (maximum disability) [19]. The MRC SS is calculated by applying the Medical Research Council (MRC) 5-point system (0 = paralysis; 5 = normal strength) bilaterally to six muscles (upper arm abductors, elbow flexors, wrist extensors, hip flexors, knee extensors, foot doral flexors) and adding the scores of each individually assessed muscle. It ranges from 0 (minimum strength) to 60 (maximum strength) [20].

Neither approval of the institutional review board nor patient informed consent were required according to the local ethics committee of the university hospital Bonn due to the retrospective character and the analysis of anonymized patient records. All study protocols and procedures were conducted in accordance with the Declaration of Helsinki.

### 2.2. Statistical Analysis

The distribution of the data was analyzed with histograms and the Shapiro’s test. Differences between groups were tested with a Student’s *t*-test or with a Wilcoxon’s test.

Association of IVIg dose was assessed with univariable regression and multivariable regression analyses. The univariable and multivariable logistic regression analyses were performed with the following prespecified variables: age, sex, diabetes, obesity, MGUS, clinical course of CIDP (relapsing, initial manifestation as GBS-like), INCAT score, MRC SS, delay between symptom onset/diagnosis, disease duration, and CSF protein. The best performing multivariable regression model was selected based on its highest r-squared value and on the relatively smallest Akaike information criterion. The calculations were performed with R, version 4.0.3 (R Core Team, 2019, Vienna, Austria).

## 3. Results

**Figure 2:**
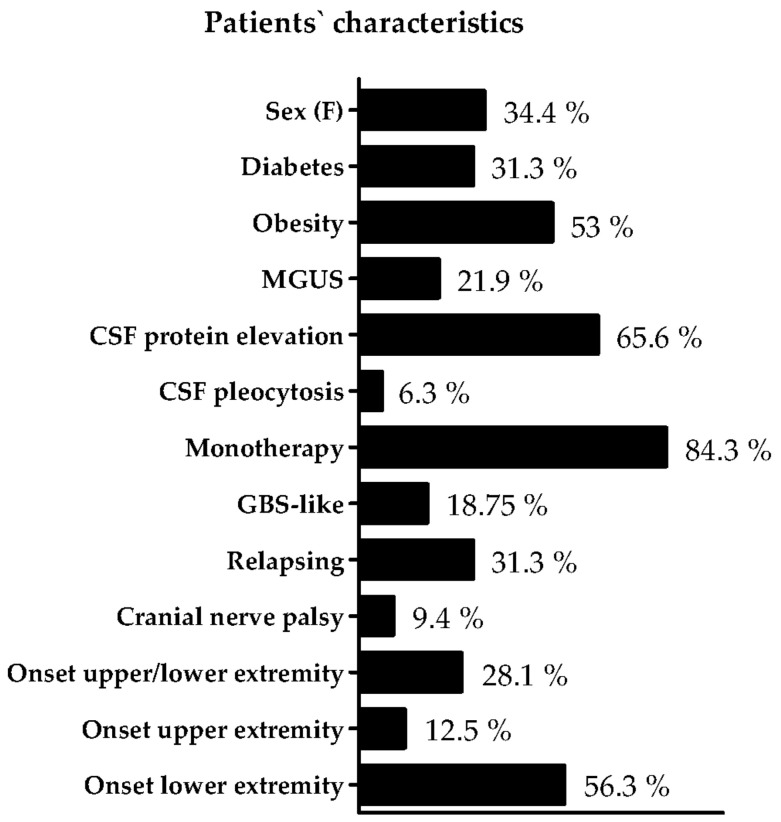
Individual patients’ characteristics. An overview of the characteristics of the CIDP patients is presented. Abbreviations: MGUS monoclonal gammopathy of undetermined significance, GBS Guillain–Barre Syndrome, CSF Cerebrospinal fluid.

The mean IVIg dose in our cohort was 1.07 ± 0.37 g/kg in four weeks. A detailed depiction of the individual patients’ characteristics can be found in Figure 2 and Table 1. Median age of the patients was 64 years (IQR 57–70). 11 patients (34.4%) were female. 10 patients (31.3%) were diagnosed with diabetes, 17 had obesity (53.1%), and, in seven patients, MGUS was detected (21.9%). The results of the lumbar puncture performed when the diagnosis was made was evaluated. 21 patients (65.6%) had elevated protein level in the CSF. Two patients (6.3%) had a pleocytosis in the CSF. 27 patients (84.3%) were treated with IVIg as monotherapy. Onset of GBS-like symptoms was recorded in six patients (18.8%), and a relapsing form was detected in 10 patients (31.3%). Three patients (9.4%) had affection of cranial nerves during the disease course. Nine patients (28.1%) had affection of both lower and upper extremity as initial manifestation. Only the upper extremity was initially symptomatic in four patients (12.5%), and only the lower extremity was initially symptomatic in 18 patients (56.3%). The median time of disease duration was 10 years (IQR 4.8–14.3), and the delay between symptom onset and diagnosis of CIDP was one year (IQR 0–3.5). Mean INCAT score was 2.8 (median 2, IQR 1–4.6), and mean MRC SS was 55.8 (median 58, IQR 53.8–60).

Age, disease duration, time between onset and diagnosis, and lower MRC SS was highly significantly associated (*p* < 0.001) with the required IVIg dose. CSF protein elevation and a higher INCAT score was significantly associated (*p* < 0.05) with the required IVIg dose.

A detailed depiction of the results of the univariable regression analyses can be found in Table 2. The coefficient for age was 0.01 (95% coefficient interval 0.0–0.02, *p*-value 0.018), the coefficient for CSF protein elevation was 0.42 (95% coefficient interval 0.09–0.74, *p*-value 0.0141), and the coefficient for the INCAT score was 0.07 (95% coefficient interval 0.01–0.12, *p*-value 0.014). The coefficient for the MRC SS score was −0.03 (95% coefficient interval −0.05–0.0, *p*-value 0.027). No significant correlations between the other factors and the drug dose in the univariable regression analysis was detected.

**Figure 3:**
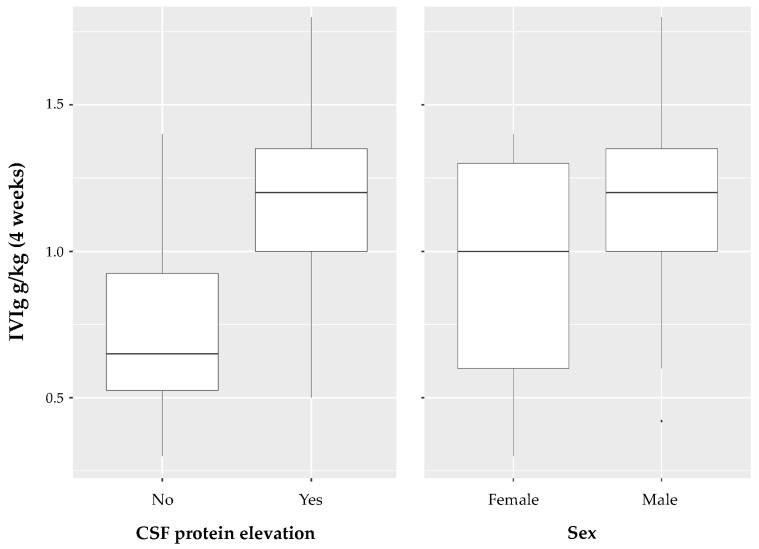
Box plot diagram for CSF protein elevation and sex. For the two characteristics (CSF protein elevation and sex) with the highest coefficients in the multivariabe regression model, a box plot diagram depicting the pattern of data distribution is shown.

The multivariable regression model that incorporated age, sex and CSF protein elevation, the MRC SS, and the time interval between onset and diagnosis achieved the relatively lowest Akaike information criterion of all multivariable linear regression models with an adjusted R-squared value of 0.43 (Table 2). For the two characteristics (CSF protein elevation and sex) with the highest coefficients in the multivariable regression model, a box plot diagram depicting the pattern of data distribution is shown in Figure 3.

## 4. Discussion

CIDP is a chronic, immune-mediated neuropathy [21,22]. In one study, over 50% of CIDP patients had been unable to lead an independent existence at some stage during the disease [23]. However, the majority of CIDP patients remain stable with therapy [23].

Despite its strong evidence for the treatment of CIDP and the low incidence of side effects, finding the optimal dose of IVIg for individual CIDP patients remains challenging [12,13,14,15]. Considering the shortage of IVIg we had recently experienced, the high health-care costs of IVIg therapy and, furthermore, the reduced independence of the patients caused by treatment administration settings, an evaluation which patients can be reduced faster and which factors are more likely to lead to a higher IVIg dose is extremely important. In addition, many patients are hesitant to reduce their IVIg dose due to concerns about potential clinical worsening. Therefore, it is important to identify factors that can justify faster dose reduction.

The dose of IVIg depends primarily on the activity of the disease. Predominantly, reducing or increasing the dose is based on the clinical course. However, beyond that, there is only limited evidence, and individual parameters are associated with a higher IVIg dose in stable patients. Thus, we suggest factors associated with required drug dose. Age, CSF protein elevation, disease duration, delay between symptom onset/diagnosis, and clinical scores (INCAT score and MRC SS) were significantly associated with the required IVIg dose in our study. The best performing multivariable model incorporated age, sex, CSF protein elevation, MRC SS, and the time interval between symptom onset and diagnosis. These factors were linearly related to the drug dose, whereas the models that incorporated the other factors performed worse. Older patients, those of male gender, and patients with lower MRC SS and longer time interval between symptom onset and diagnosis appear to require higher doses of IVIg.

There are several studies which identified factors associated with IVIg treatment response. Thirouin et al. 2022 showed an elevated protein concentration in the CSF predicted therapeutic response [24]. In addition to abnormal CSF, other prognostic factors, such as relapsing–remitting course and female gender, showed an independent positive factor for treatment response. Therapeutic response was assessed after six months of treatment in this study, and they used several scales validated for inflammatory neuropathies to evaluate treatment response. Treatment of CIDP also appears to be less effective in patients with longer disease course, chronic progressive CIDP, and slow progression of symptoms in another study [24,25]. The patients with chronic progressive CIDP also tended to be older in this study. Furthermore, CIDP patients were more likely to benefit if there was a shorter time interval between disease onset and start of the treatment [26]. Although these results address all the factors we found to be associated with the required IVIg dose, the discussed studies evaluate factors related to treatment response in CIDP patients, but they do not evaluate factors that are related to the IVIg dose in stable patients. It is likely that there is an association between initial treatment response and IVIg dose in stable patients. Therefore, these results might further support our conclusions.

There is another study analyzing factors associated with the required drug dose in IVIg maintenance treatment. Rajabally et al. analyzed 15 patients with CIDP in this study. In contrast to our study, they did not find a correlation between IVIg dose and factors they analyzed, such as weight, disease duration, or pre-therapeutic degree of disability [27]. This could be due to the small number of patients included in the study. In addition, we analyzed more parameters in our study.

In a recent study by Alonge et al., the efficacy of SCIg treatment in CIDP was evaluated. It was shown that preservation of nerve function in CIDP under treatment could be assessed using nerve conduction studies in this study [28]. In addition, this study showed that electrophysiology variables could hold a role as prognostic factors to estimate treatment efficacy and duration time [28]. In line with that, decreased compound muscle action potential and muscle atrophy indicative of axonal dysfunction seem to be pronounced in non-responders of IVIg, also leading to a worse long-term prognosis [25]. The axonal degeneration process is associated with diagnostic and therapeutic delay [29]. Axonal loss, in contrast to pure demyelination, is more likely to be irreversible.

In addition to the parameters discussed here, we also found an association of IVIg dose with a higher INCAT score and a lower MRC SS. These patients may be more vulnerable to deterioration due to reduced resources. This might lead to higher dosage of IVIg.

Up to now, there are no established biomarkers available to assess the therapeutic effect in CIDP during IVIg therapy. However, a recent study showed the plasmatic pattern of matrix metalloproteinases (MMPs), especially MMP-9 and MMP-2 and their physiological tissue inhibitors, which are markedly altered in patients with CIDP [30]. Plasma neurofilament light chain (pNFL) might be another possible biomarker in CIDP [31]. pNfL was higher in CIDP patients before IVIg treatment initiation than in healthy controls, it was subsequently decreased, and it was comparable to the control after IVIg induction. In addition, among CIDP patients under IVIg treatment, pNfL concentration was significantly higher in unstable patients than in stable patients. There was a statistically significant correlation between pNfL concentration at time of IVIg withdrawal and the likelihood of relapse. Sphingomyelin or neural cell adhesion molecule are other biomarkers that have been studied for CIDP [32]. Further studies will show whether these parameters can also be used to monitor response and dosing in patients with CIDP treated with IVIg.

Finally, variability in pharmacokinetics and bioavailability can affect responsiveness of IVIg in individual CIDP patients [2]. GBS patients show a large variability in serum immunoglobulin G (IgG) levels after standard IVIg treatment in one study. In addition, the increase in IgG after administration was associated with clinical outcome in GBS patients [33]. A high variability of IgG levels after IVIg treatment was also reported for CIDP patients, although the association with treatment response is not clear [34]. Several genetic variations have been linked to responsiveness to IVIg.

Taken together, a variety of factors appears to be associated with therapy response. In addition, our study reveals additional factors that are associated with the required IVIg dosing.

Limitations of our study are the retrospective design and the small group size. In addition, the CIDP patients in our study were relatively mildly affected. Thus, larger and prospective studies would be important to confirm the results in our study.

## 5. Conclusions

Our model, which consists of routine parameters that are simple to address in clinical practice, can assist in individual dose finding in stable CIDP patients. Older patients, those of male gender, patients with lower MRC SS, and those with a longer time interval between symptom onset and diagnosis appear to require higher doses of IVIg. These data can help to adjust the dose of IVIg more precisely and more rapidly in stable CIDP patients.

## Figures and Tables

**Table 1:** Patients’ characteristics and association with drug dose.

		Drug Dose, *p*-Value
Age, median, (iqr)	64 (57–70)	<0.001 **
Sex, female *n*, (%)	11 (34.4)	0.207
Diabetes, *n*, (%)	10 (31.3)	0.350
Obesity, *n*, (%)	17 (53)	0.358
MGUS, *n* (%)	7 (21.9)	0.183
MGUS IgM	3 (9.4)	0.631
MGUS IgA	2 (6.3)	-
MGUS IgG	4 (12.5)	0.110
CSF protein elevation, *n*, (%)	21 (65.6)	0.049 *
CSF pleocytosis, *n*, (%)	2 (6.3)	-
IVIg monotherapy, *n*, (%)	27 (84.3)	0.107
GBS like, *n*, (%)	6 (18.75)	0.147
Relapsing, *n*, (%)	10 (31.3)	0.412
Cranial nerve palsy, *n*, (%)	3 (9.4)	0.909
Upper and lower extremity, *n*, (%)	9 (28.1)	0.606
Only upper extremity, *n*, (%)	4 (12.5)	0.455
Only lower extremity, *n*, (%)	18 (56.3)	0.705
Disease duration (median, iqr)	10 (4.8–14.3)	<0.001 **
Delay onset/diagnosis (median, iqr)	1 (0–3.5)	<0.001 **
INCAT Score, median (iqr)	2 (1–4.6)	0.013 *
MRC SS, median (iqr)	58 (53.8–60)	<0.001 **

A highly significant association (**, *p* < 0.001) with the required intravenous immunoglobulins (IVIg) drug dose was detected for age, disease duration, Inflammatory Neuropathy Cause and Treatment (INCAT), and Medical Research Council Sum Score (MRC SS). A significant association (*, *p* < 0.05) with the last documented drug dose was found for cerebrospinal fluid (CSF) protein elevation and delay between onset and diagnosis. The other parameters showed no significant findings.

**Table 2:** Uni- and multivariabel model.

	Univariable Model		Multivariable Model	
	Coefficient	*p*-Value	Coefficient	*p*-Value
Age	0.01 (0.00–0.02)	0.0175 *	0.01 (0.0–0.02)	0.149
Sex, male	0.19 (−0.09–0.02)	0.182	0.32 (0.02–0.62)	0.037
CSF protein elevation	0.42 (0.09–0.74)	0.0141 *	0.33 (0.02–0.65)	0.036
Diabetes	0.11 (−0.18–0.40)	0.437		
Obesity	0.12 (−0.15–0.39)	0.356		
MGUS	0.23 (−0.09–0.55)	0.157		
GBS like	−0.22 (−0.56–0.13)	0.214		
Relapsing	−0.1 (−0.4–0.19)	0.483		
INCAT score	0.07 (0.01–0.12)	0.014 *		
MRC SS	−0.03 (−0.05–0.00)	0.027 *	−0.02 (−0.05–0.0)	0.080
Delay onset/diagnosis	0.01 (−0.01–0.04)	0.303	−0.02 (−0.05–0.01)	0.172

Depiction of the results of the univariable and multivariable regression analyses. Depicted are the coefficients, 95% coefficient intervals, and the *p*-values for the univariable models and the best performing multivariable regression model. A significant correlation (*, *p* < 0.05) in the univariable regression analysis was found for age, CSF protein elevation, INCAT score, and MRC SS. No significant correlation between the other factors and the drug dose in the univariable regression analysis was detected. The multivariable regression model that incorporated age, sex, and CSF protein elevation, as well as the MRC SS and the time interval between onset and diagnosis achieved the relatively lowest Akaike information criterion of all multivariable linear regression models with an adjusted R-squared value of 0.43.

## Data Availability

The data presented in this study are available upon reasonable request from the corresponding author.
